# The Effect of Risk Prevention Ability on Entrepreneurial Performance of Chinese College Students: Moderating Effect of Team Management Ability

**DOI:** 10.3389/fpsyg.2022.861929

**Published:** 2022-04-22

**Authors:** Yuting Zhu, Shaowei Qu, Hebo Jin, Zhaohui Li

**Affiliations:** ^1^School of Economics and Management, University of Science and Technology Beijing, Beijing, China; ^2^Institute of Educational Economics and Management, University of Science and Technology Beijing, Beijing, China

**Keywords:** risk prevention ability, team management ability, entrepreneurial performance, college students, moderation

## Abstract

Improving the performance and success rate of college students’ new ventures has attracted increasing attention globally. In this study, a questionnaire survey was conducted among 1500 college students who were directly involved in entrepreneurial activities in 23 provinces in China. The study explores the effects of team management and risk prevention abilities on college students’ entrepreneurial performance. The results show that risk prevention ability significantly increases university students’ entrepreneurial performance (e.g., profit and loss status, capital flow, and staff flow). Team management ability enhances these entrepreneurial indicators to varying degrees, with a “threshold” effect of its impact on entrepreneurial performance. With a continued increase in team management ability, the effect of risk prevention ability on entrepreneurial performance becomes increasingly more significant. Specifically, when team management ability reaches a medium level and above, risk prevention ability significantly improves capital flow and staff stability; and when team management ability reaches a high level, risk prevention ability significantly improves enterprise profitability indicators.

## Introduction

In the wake of COVID-19, China’s job market has been sluggish, while the number of college graduates in China has been rising. An important measure to resolve this contradiction is to strengthen entrepreneurship education (Long et al., 2021). The goal of entrepreneurship education is to equip students with entrepreneurial consciousness and ability and to train them to think innovatively. From the perspective of social value realisation, the purpose of entrepreneurship education is to raise the survival rate of college students’ entrepreneurial enterprises; the generation and improvement of college students’ entrepreneurial ability is an important objective symbol of improved entrepreneurial performance. At present, college students’ entrepreneurship in China is characterised by the “double low” situation of low participation and low success rates. How college students’ entrepreneurship performance can be enhanced is one of the key factors in ensuring the improvement of the “two low rates.”

Regarding the nature of entrepreneurial performance, there are arguments about “behaviour” and “result” in the academic circle (Cai and Zhou, 2008). Haber and Reichel (2005) defined entrepreneurial performance as the synthesis of short-term and long-term profits reflecting the operating conditions of enterprises from the perspective of process. Venkataraman (2004) defined entrepreneurial performance as the entrepreneurial achievement to realise economic benefits. Yang (2020) considered the results and benefits generated in the transformation of entrepreneurial ability into entrepreneurial practice from the perspective of performance results. In present study, mature enterprises’ performance is generally measured by return on total assets (Zhao and Sun, 2015), sales growth rate, return on investment, and market share, etc. The measurement of entrepreneurial performance is generally based on corporate financial indicators (Venkataraman, 2004) and non-financial indicators, such as corporate development prospects and service quality (Anderson and Sullivan, 1993). However, new ventures must first think about profit and survival (Xu et al., 2020). For survival, an enterprise’s survival time and overall life are measured (Ciavarella et al., 2004). There are also indicators that are based on the three dimensions of survival, growth, and innovation for measuring entrepreneurial performance (Zhan et al., 2020), including net income, sales, capital flow, market share, an enterprise’s operating time, and survival hopes (Ciavarella et al., 2004).

We contend that college and university students’ new ventures belong to the category of start-up and small–micro enterprises, and have distinct characteristics, such as short start-up time, financing difficulties, lack of operational experience and social capital, shortage of market resources, conflicts between entrepreneurship and academic progress, and even immature technology, as well as many other problems and challenges. Measurement of entrepreneurial performance should not be based on social enterprises’ financial indicators, but should focus more on the results achieved in the entrepreneurial process. In other words, the measurement should focus on “entrepreneurial process performance” and should emphasise entrepreneurial consciousness, spirit, and ability, as well as college students’ personal experiences. The accumulation of this human capital will become a key factor in future career development. College students’ entrepreneurial performance is the sum of stable operation, rational utilisation of resources, and periodic profits achieved in the entrepreneurial process.

The realisation of entrepreneurial performance is closely related to entrepreneurial ability. Entrepreneurship is a composite concept with many factors. Timmons et al. (1999) proposed that entrepreneurship was a highly dynamic process with the joint action of the three elements of opportunity, resources, and team. Therefore, scholars have actively discussed the relationship between entrepreneurial ability and entrepreneurial performance, including opportunity identification ability, resource integration ability, and team management ability (Sang et al., 2012; Chen and Chen, 2016; Yang et al., 2019). Chandler and Hanks (1994) verified the positive impact of opportunity identification and resource integration on entrepreneurial performance.

In the context of Chinese society, half of the college students’ start-ups are small enterprises with a scale of 5–10 employees and are different from ordinary social entrepreneurship. (1) The enterprises are mainly start-ups with a short life cycle. (2) The enterprise scale mainly consists of small and micro enterprises, with limited start-up capital, few fixed assets, and small teams. (3) The business model mainly focuses on the replication and improvement of traditional industries; most of them start their businesses for the purpose of “employment,” with few business model innovations. (4) The commercial added value of the enterprise products, the product maturity, and the technology content is low. (5) The financing channels are narrow, and the financing scale is relatively small. (6) In terms of time, there is a conflict between entrepreneurship and study, the latter crowding out the time and energy for learning the professional knowledge and skills of entrepreneurs. College students’ entrepreneurial enterprises are small, with a short life cycle and low product maturity; these characteristics pose challenges to the enterprises’ survival and are unfavourable factors that give rise to many potential risks that directly influence or threaten the success of college students’ entrepreneurship. Therefore, risk prevention management ability is one of the key factors in ensuring the survival of college students’ venture enterprises. Botha and Robertson (2014) argued that successful entrepreneurs possessed higher risk management capabilities. Successful enterprise risk management can effectively enhance an enterprise’s value (Lechner and Gatzert, 2018), can smooth its income fluctuations, can reduce the impact of financial crises, and can improve its performance (Ashraf et al., 2017).

There is an interdependent relationship between entrepreneurial ability and performance (Mitchelmore and Rowley, 2010; RezaeiZadeh et al., 2017). For Chinese college student entrepreneurs, team management ability is an important ability for maintaining the operation of their enterprises. On the one hand, college students’ entrepreneurial enterprises have small teams, with friendship the main emotional connection; thus, it is critical to maintain team members’ participation in the construction of entrepreneurial enterprises. On the other hand, from the perspective of human capital, effective team management ability can help bring members’ diversity into play, can highlight each team member’s entrepreneurial advantages, and can avoid or reduce the probability of entrepreneurial risks from different dimensions. The team members of college students’ new ventures are typically schoolmates. Friendship and trust are the main factors in maintaining the stability of the team. Francis and Sandberg (2000) verified the effect of friendship on entrepreneurial teams and demonstrated that team building based on friendship could improve corporate performance. For college students’ new ventures, the essence of friendship is team management. Students’ new ventures are collaborative teams. Team members can maximise cohesion, tolerance, cooperation, and solidarity to establish a management power unit based on friendship, trust, morality, and ability recognition. This kind of management power ensures that new ventures can integrate resources, information, capital, market, and other links in the operation process sufficiently well to maximise benefits in effective, tacit, and active cooperation.

Studies have analysed the effects of social networks (Sun and Wei, 2021), network embedding (Yang et al., 2020), opportunity innovation (Chen et al., 2019), entrepreneurial motivation (Xu and Chen, 2017), entrepreneurial orientation (Ren et al., 2017a), and proactive personality (Ren et al., 2017b) on entrepreneurial performance to improve college students’ entrepreneurial performance. Scholars have also discussed the relationship between entrepreneurship education (An and Zhang, 2016), entrepreneurial capital (He and Song, 2015), entrepreneurial ability (Yang et al., 2019), and entrepreneurial performance. However, college students’ risk prevention and team management abilities and their interaction have rarely been discussed. Therefore, this study focuses on the effect of college students’ risk prevention ability on their entrepreneurial performance and how team management ability affects the entrepreneurial performance through effective risk prevention, to explore the relationship between risk prevention and entrepreneurial performance. The study explores how the success rate of college students’ entrepreneurship can be improved to provide a scientific basis for improving the effectiveness of entrepreneurship education in colleges and universities.

## Hypotheses

### The Influence of College Students’ Risk Prevention Ability on Entrepreneurial Performance

Risk management is the core competence of enterprise competitiveness and a core factor in enterprises’ internal systems control (Yang et al., 2018). Understanding, managing, and avoiding risk are important ways for entrepreneurs to reduce business failure. Risk refers to the probability of loss and significance of commercial risk (Mitchell, 1995). Vaughan and Vaughan (2001) believed that risk management included risk identification, risk quantification and evaluation, and risk management and control to continuously report on risk developments. Entrepreneurs who have the ability to understand, manage, and avoid risks can improve their business performance and increase their income (Okolie et al., 2021). The operation of entrepreneurial enterprises is a result of enterprise operation and capital flow adequacy (Padachi, 2006). Clarke and Varma (1999) believed that risk management was a strategic process and that the ultimate goal of risk prevention pretreatment was to ensure that enterprises achieved sustainable returns. Regarding the relationship between risk management and performance, the existing studies mainly focus on listed enterprises (Mohammed and Knápková, 2016), such as the positive impact of risk management on enterprises’ new product development performance (Mu et al., 2009). In addition, relevant empirical studies focus on financial institutions (Callahan and Soileau, 2017), that is, to increase the profitability of a company by reducing different operating and marginal costs and reducing the uncertainty of stock market returns (Eckles et al., 2014).

Enterprise risk management can reduce different types of costs associated with enterprises’ operational and non-operational activities (Khan et al., 2016), as well as different types of risk disclosure (Florio and Leoni, 2016). There is no doubt that risk management plays a significant role in improving mature enterprises’ performance. The question is whether this effect exists in new ventures, especially college students’ new ventures. Yang (2020) employed a logistic regression model to explore the impact of graduates’ entrepreneurial ability factors on entrepreneurial performance and found that risk prevention had the most significant impact of all entrepreneurial ability factors. Therefore, ensuring an enterprise’s smooth operations requires risk prevention ability. In particular, college students’ new ventures are vulnerable to risk threats, as they are mainly small- and micro-scale start-ups with low customer stickiness due to low product maturity. In this study, the risk prevention ability of college students’ new ventures refers to the comprehensive ability of enterprises to realise, predict, control, and dispose of risks. The hypotheses that follow are proposed based on the above analysis.

H1: *Risk prevention ability can improve the entrepreneurial performance of college students’ new ventures.*

### The Influence of College Students’ Team Management Ability on Entrepreneurial Performance

An enterprise’s leadership establishes the team management and decides the enterprise’s development direction. The complexity of internal processes is positively correlated with the complexity of entrepreneurial activities. Individuals often cannot manage entrepreneurial activities; thus, the establishment and management of an entrepreneurial team have become an indispensable ability for entrepreneurs. Team management is different from leadership, which is the ability to inspire and guide followers to achieve their goals (Renko et al., 2015). Team management ability is an important quality for entrepreneurs to unite internal staff, ensure smooth communication among employees, and achieve stable operations. It is also the embodiment of team cohesion.

The role of an entrepreneurial team includes leading, organising, and communicating. The first step for college students is to establish an entrepreneurial team. The simple sum of team members’ human capital may not affect entrepreneurial performance; however, the heterogeneity of the entrepreneurial team does (Jin et al., 2017). Chen and Chen (2016) verified the mediating effect of entrepreneurial team characteristics on an enterprise’s financial, market, and competitive performance. College students’ entrepreneurial team members suffer from a lack of work experience and slow transformation of knowledge to skills. The natural weakness of team members is bound to affect team behaviour and, therefore, enterprise performance (Hu et al., 2017). To build a heterogeneous entrepreneurial team, it is necessary for college students to be able to manage teams. The impact of team leadership on entrepreneurial performance has been demonstrated (Knipfer et al., 2018; Miao et al., 2019). However, in a complex entrepreneurial environment, team members must communicate fully to ensure that they give full play to their innovative talents (Hart, 2014; Zhou, 2016). Chen et al. (2020) believed that it was difficult to achieve this through team leadership. By proposing the concept of shared leadership, they demonstrated that shared team leadership could improve entrepreneurial performance by giving full play to the leadership ability of each entrepreneurial team member, conducting team self-reflection, and crossing teams. El-Awad et al. (2017) regard a team as an information processor, and a timely and accurate information processing ability can promote performance. Meanwhile, self-regulating teams improve corporate performance by adjusting team behaviour and operation mode; however, this process is challenging (Forsstrom-Tuominen et al., 2019). Leadership plays a positive role in promoting information processing ability (Knipfer et al., 2018) and realising team self-regulation (Lyubovnikova et al., 2017), while shared leadership (Chen et al., 2020) also plays a positive role. For college student entrepreneurs, leadership means playing the leading role in the entrepreneurial team, while shared leadership means playing the communication and coordination role in the entrepreneurial team; however, both are within the scope of team management ability. That is, enterprises with high levels of team management ability are characterised by effective integration of resources, rapid identification of opportunities, and smooth communication of information, and thus continuously improve their performance. The hypotheses that follow are proposed based on the above analysis.

H2: *Team management ability can improve the performance of college students’ new venture.*

### Moderating Effect of Team Management Ability

When faced with uncertainty in a turbulent market, management thinking and behaviour will affect enterprise risk management practice. Therefore, to improve enterprise performance, both enterprise risk management ability and some internal management ability are required (Arena et al., 2010). The team management ability of college students’ new ventures is a key factor in preventing and controlling the risk of enterprise operation. Team management ability is manifested by the cohesion of employees. When faced with risks, a team with high cohesion can effectively tackle the risks and formulate a solution to risks, thus achieving sustainable operations and revenue performance on the premise of ensuring the smooth operation of the enterprise. Existing studies have shown that entrepreneurial team heterogeneity can improve the performance of start-ups by giving play to the skill diversity of members (Finkelstein and Hambrick, 1996), but team member heterogeneity may also lead to conflict and reduce the performance of start-ups (O’Reilly et al., 1989). Adjusting team conflicts and supporting and enabling the self-regulating role of the team will help avoid the occurrence of risks (Wu et al., 2019). Following the transformation of a new venture to business model, the impact of resolving relationship conflicts on improving team performance is particularly significant (Boone et al., 2020). Strong team management ability improves the effect of risk control and management; in contrast, low team management ability will lead to the expansion of the enterprise risk coefficient.

Specifically, when there is insufficient team management ability, the channel of communication within the team is not smooth, which reduces the information transmission speed. Information asymmetry and information flow occlusion result in enterprises’ inability to discover potential risks in time. Reduced team management ability can lead to the breakdown of communication mechanism, and enterprises will be unable to control the risks quickly. Poor cooperation leads to unclear division of power and responsibility among employees and reduced team cohesion, which further intensifies internal conflicts and loosens the internal structure. Continuous internal friction among teams leads to an expansion of the enterprise risk coefficient and the generation of new risks. Risks are interdependent, and the occurrence of risk may result in the emergence of continuous risks (Venkatesh et al., 2015). For the existing risks, the chaotic team order and low-risk management ability can lead to further expansion of the risk coefficient, and an inability to effectively mitigate the impact of internal and external risks on the enterprise, threatening the enterprise’s survival. The reduced team cohesion and the loss of core team members trigger incomplete personnel allocation, which ultimately leads to a decline in enterprise performance and possibly the shutdown of the enterprise’s operation. The hypotheses that follow are proposed based on the above analysis.

H3: *Team management ability positively moderates the impact of risk prevention on entrepreneurial performance.*

## Data

### Procedure

The scale of the risk prevention ability factor was modified according to the study by Okolie et al. (2021), while that of team management ability was modified following Yang (2020). A 5-level Likert-scoring scale was adopted, with 1 denoting a low degree of compliance and 5 a high degree. Regarding the performance indicators for college students’ new ventures, this study refers to the design of entrepreneurial performance indicators in Yang’s (2020) study, as well as the financial indicators for mature enterprises and start-ups. It considers the target motivation of college students’ new ventures, namely, market cash flow and performance, to design six questions. Following the Delphi method and verification by exploratory factor analysis, three indicators were finally determined to represent entrepreneurial performance, namely, profit and loss (P&L) status (Y1), capital flow (Y2), and staff flow (Y3). Each index contained three items, as shown in Table 1.

**TABLE 1:** Questionnaire.

Indicator		Item
Risk prevention ability	A11	Have strong risk awareness and standard measures
	A12	Have preventive measures against risks in market and technology fields
	A13	Able to anticipate, control and deal with potential risks
Team management ability	A21	Able to build the core team needed to start a business
	A22	Able to ensures a high degree of division of labor among team members
	A23	Able to established an effective communication mechanism within the team
Entrepreneurial performance	Y1	What is the current profit and loss status of your enterprise
	Y2	What is the current capital flow status of your enterprise
	Y3	What is the current staff flow of your enterprise

We invited 1,500 students from general and vocational colleges in 23 provinces to participate in the survey. All questionnaires were distributed online, of which 1,467 were recalled. By reserving the choices of “whole-heartedly starting my own business” and “entering college but continuing to start my own business,” 1,090 questionnaires were obtained. To ensure the accuracy of the results, responses containing null values were removed, and 1,054 questionnaire data were finally obtained. The effective recovery rate was 70.27%.

### Simple

Based on the regional distribution characteristics of the sample, 32 were in northeast China (3.04%), 174 in eastern China (16.51%), 40 in central China (3.80%), 805 in western China (76.38%), and 3 in Hong Kong and Macao (0.28%). In the sample structure, 411 were men (38.99%) and 643 were women (61.01%). Among the acquired degrees, 222 (21.06%) students had specialised degrees, 725 (68.79%) students had bachelor’s degrees, and 107 (10.1%) students had master’s degrees. From the grades in the sample, 190 were junior students, accounting for 11.20%, 118 were senior students, accounting for 18.03%, 406 and 283 were freshmen and sophomores, accounting for 38.52% and 26.85%, respectively, and 57 were graduate students, accounting for 5.41%. There were 924 students from ordinary colleges, accounting for 87.67%, and 130 students from vocational colleges, accounting for 12.33%.

The mean values, standard deviations, and correlation results for risk prevention ability, team management ability, and entrepreneurial performance are shown in Table 2. All the indicators are positively correlated with the P&L status, capital flow, and staff flow of entrepreneurial performance.

**TABLE 2:** Descriptive statistics and correlation analysis.

	Mean	STD	A11	A12	A13	A21	A22	A23	Y1	Y2	Y3
A11	3.61	0.940	1.000								
A12	3.58	0.915	0.816**	1.000							
A13	3.59	0.938	0.814**	0.803**	1.000						
A21	3.59	0.955	0.747**	0.742**	0.723**	1.000					
A22	3.64	0.917	0.751**	0.752**	0.726**	0.815**	1.000				
A23	3.62	0.940	0.770**	0.759**	0.744**	0.791**	0.831**	1.000			
Y1	2.76	0.940	0.120**	0.107**	0.131**	0.105**	0.101**	0.127**	1.000		
Y2	2.65	1.172	0.088**	0.098**	0.072**	0.060*	0.058*	0.053*	0.345**	1.000	
Y3	2.98	1.071	0.100**	0.111**	0.110**	0.089**	0.084**	0.078**	0.263**	0.432**	1.000

*p < 0.05, **p < 0.01.

### Confirmatory Factor Analysis

Risk prevention ability (RISK), team management ability (TEAM), and entrepreneurial performance (Prfo) were all measured on scales. Cronbach’s α coefficients for risk prevention ability and team management ability were both greater than 0.9, indicating good reliability of the scale. Cronbach’s α coefficient for entrepreneurial performance was 0.589, close to 0.6, indicating that the reliability of the entrepreneurial performance scale was acceptable. Confirmatory factor analysis is required before the construction of the mediation effect, and the factor loading is shown in Table 3. The model results showed that chi-square/degree of freedom (χ^2^/*df*) = 39.184/24 = 1.633 < 3, root mean square error of approximation (RMSEA) = 0.025 in the range of [0.009, 0.038], comparative fit index (CFI) = 0.998 > 0.9, Tucker-Lewis index (TLI) = 0.996 > 0.9, and standardized residual mean root (SRMR) = 0.020 < 0.08. The model fits well. The factor loadings for risk prevention ability and team management ability are both greater than 0.8; the construct reliability (CR) value is greater than 0.7, while the average variance extracted (AVE) value is greater than 0.5. The entrepreneurial performance factor loading is greater than 0.4, the CR value is greater than 0.6, and the AVE value is 0.346, indicating that the model is acceptable. The AVE square root values are larger than the index correlation coefficient, and the model has good aggregation and discriminant validities.

**TABLE 3:** Factor load and reliability and validity test.

	Cronbachs’α	Estimate	CR	AVE	RISK	TEAM	Prfo
RISK	0.920	0.875–0.897	0.918	0.790	**0.888**		
TEAM	0.918	0.876–0.903	0.921	0.795	0.775	**0.891**	
Prfo	0.589	0.401–0.695	0.603	0.346	0.100	0.151	**0.588**

*The diagonal bold values are the AVE square root.*

## Empirical Results

### Model Results

Using entrepreneurial performance, P&L status, capital flow, and staff flow as the dependent variables, and “RISK,” “TEAM,” and “RISK × TEAM” as the independent variables, the impacts of risk prevention and team management abilities on each index of college students’ entrepreneurial performance were evaluated. The results are shown in Table 4.

**TABLE 4:** Results of models.

	Entrepreneurial performance			P&L status (Y1)		
	Model 1.1	Model 2.1	Model 3.1	Model 1.2	Model 2.2	Model 3.2
RISK	0.069**	0.177**	0.183**	0.126**	0.140	0.144
	(2.923)	(2.778)	(2.827)	(3.406)	(1.182)	(1.227)
	[0.023, 0.115]	[0.052, 0.302]	[0.056, 0.310]	[0.053, 0.198]	[−0.092, 0.372]	[−0.086, 0.374]
TEAM		−0.133	−0.086		−0.019	0.014
		(−1.872)	(−1.677)		(−0.141)	(0.146)
		[−0.272, 0.006]	[−0.186, 0.014]		[−0.278, 0.241]	[−0.180, 0.209]
RISK × TEAM			0.053**			0.098***
			(2.680)			(3.898)
			[0.014, 0.093]			[0.049, 0.147]

	**Capital Flow (Y2)**			**Staff Flow (Y3)**		
	**Model 1.3**	**Model 2.3**	**Model 3.3**	**Model 1.4**	**Model 2.4**	**Model 3.4**

RISK	0.111*	0.388	0.393**	0.134**	0.325*	0.330*
	(2.147)	(0.274)	(2.582)	(3.018)	(2.120)	(2.160)
	[0.010, 0.212]	[−2.393, 3.169]	[0.095, 0.691]	[0.047, 0.221]	[0.025, 0.626]	[0.031, 0.630]
TEAM		−0.335	−0.229		−0.233	−0.150
		(−0.217)	(−1.771)		(−1.363)	(−1.161)
		[−3.371, 2.700]	[−0.483, 0.024]		[−0.568, 0.102]	[−0.403, 0.103]
RISK × TEAM			0.088*			0.094**
			(2.166)			(2.918)
			[0.008, 0.167]			[0.031, 0.158]

*p < 0.05, **p < 0.01, ***p < 0.001, lower and upper bound of 2.5% confidence interval are in square brackets.

Risk prevention ability positively promotes entrepreneurial performance. The results of Model 1.1 show that the influence coefficient of risk prevention on college students’ entrepreneurial performance is 0.069, which is significant at 1%. Model 1.2 shows that risk prevention has a positive and significant effect on P&L status (β = 0.126, *P* < 0.01). Model 1.3 shows that risk prevention has a positive effect on capital flow (β = 0.111, *P* < 0.05), while Model 1.4 corroborates the positive correlation between risk prevention and staff flow (β = 0.134, *P* < 0.01); thus, H1 is supported. When team management was introduced into the model, the results of Models 2.1–2.4 showed that risk prevention still had significant effects on entrepreneurial performance (β = 0.177, *P* < 0.01) and staff flow (β = 0.325, *P* < 0.05) but no significant influence on P&L status (β = 0.177) and capital flow (β = 0.388). However, the impact of team management on entrepreneurial performance and each subindex was not significant; thus, H2 was not supported. Therefore, it is inferred that team management has no direct effect on entrepreneurial performance.

Team management strengthens risk prevention to improve entrepreneurial performance. Further analysis of the moderating effect of team management showed positive and significant influence coefficients on entrepreneurial performance and all indicators. Models 3.1–3.4 show that the 2.5% interval value for each coefficient does not include 0; that is, team management strengthens the effect of risk prevention on entrepreneurial performance.

**FIGURE 1:**
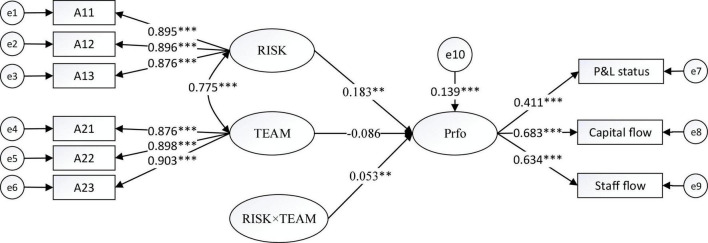
Moderating effect of TEAM on Prfo. ^**^p < 0.01, ^***^p < 0.001.

**FIGURE 2:**
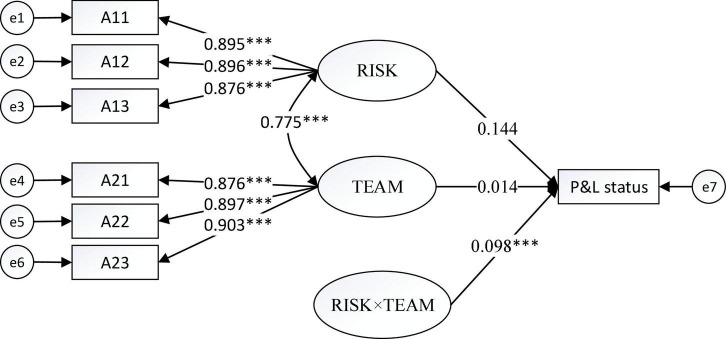
Moderating effect of TEAM on P&L. ^***^p < 0.001.

**FIGURE 3:**
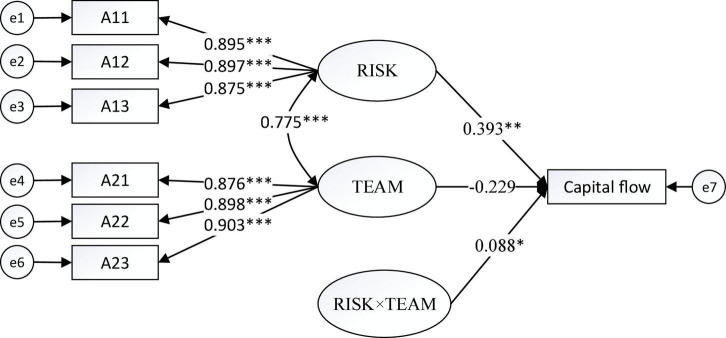
Moderating effect of TEAM on capital flow. *p < 0.05, ^**^p < 0.01, ^***^p < 0.001.

**FIGURE 4:**
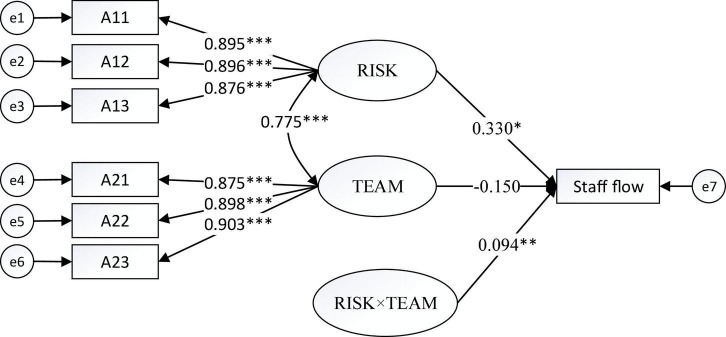
Moderating effect of TEAM on staff flow. *p < 0.05, ^**^p < 0.01, ^***^p < 0.001.

Figures 1–4 show the moderating effects of team management ability on entrepreneurial performance, P&L status, capital flow, and staff flow.

In Figure 1, the non-standardised influence coefficient of “RISK × TEAM” on Prfo is 0.053, which is significant at 1%; moreover, the influence coefficient of risk prevention on Prfo is 0.183, which is significant at 1%. Therefore, team management can positively moderate the impact of risk prevention on entrepreneurial performance, thus supporting H3.

In Figure 2, the non-standardised influence coefficient of “RISK × TEAM” on P&L status is 0.098, which is significant at 0.1%, thus suggesting a positive influence of team management on risk prevention in enhancing P&L status.

In Figure 3, the non-standardised influence coefficient of “RISK × TEAM” on capital flow is 0.088, which is significant at 5%. Furthermore, the influence coefficient of risk prevention on capital flow is 0.393, which is significant at 1%. Thus, team management promotes the impact of risk prevention on capital flow.

In Figure 4, the non-standardised influence coefficient of “RISK × TEAM” on staff flow is 0.094, which is significant at 1%. Furthermore, the influence coefficient of risk prevention on staff flow is 0.330, which is significant at 5%. Team management can thus positively moderate the impact of risk prevention on staff flow.

In conclusion, risk prevention ability significantly improved the entrepreneurial performance of college students’ new ventures. The higher the risk prevention and management ability, the better the P&L status, the more abundant the capital flow, and the more stable the staff structure. Team management ability does not directly improve the entrepreneurial performance of college students’ new ventures but positively strengthens the risk prevention ability and thus indirectly improves the entrepreneurial performance, P&L status, capital flow, and staff flow.

### Simple Slope Test

**FIGURE 5:**
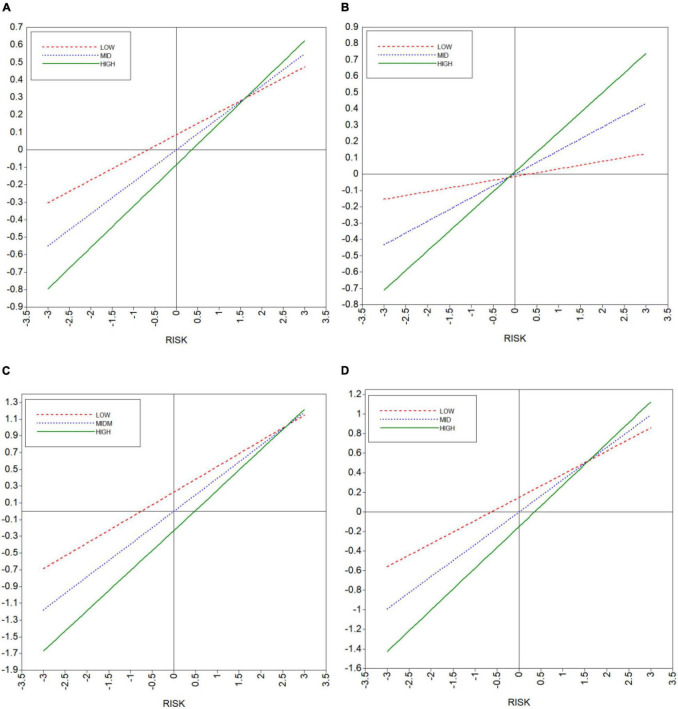
Simple slope test. **(A)** Prfo. **(B)** P&L status. **(C)** Capital flow. **(D)** Staff flow.

The selection point method is used to test whether the slope of the impact of college students’ risk prevention ability on entrepreneurial performance is different for different team management abilities. The results are shown in Figure 5. As can be seen, with an improvement in team management ability, the slopes of the impacts of risk prevention ability on entrepreneurial performance, P&L status, capital flow, and staff flow also show a gradual upward trend.

The simple slope test results of each model are shown in Table 5. In the table, “LOW” means one standard deviation of team management below the mean, “MED” means the mean, and “HIGH” means one standard deviation above the mean.

**TABLE 5:** Results of simple slope test.

TEAM	Point estimation				Confidence interval	
	Non-std estimates	S.E.	Est./S.E.	*p*-value	Low 2.5%	Upper 2.5%
**a. Entrepreneurial performance**						
LOW	0.130	0.063	2.408	0.041	0.006	0.254
MID	0.183	0.065	2.827	0.005	0.056	0.310
HIGH	0.237	0.072	3.286	0.001	0.095	0.378

**b. P&L Status**						
LOW	0.046	0.120	0.388	0.698	–0.188	0.281
MID	0.144	0.117	1.227	0.220	–0.086	0.374
HIGH	0.242	0.121	2.004	0.045	0.005	0.478

**c. Capital Flow**						
LOW	0.305	0.159	1.925	0.054	–0.006	0.616
MID	0.393	0.152	2.582	0.010	0.095	0.691
HIGH	0.480	0.156	3.075	0.002	0.174	0.787

**d. Staff Flow**						
LOW	0.236	0.155	1.521	0.128	–0.068	0.541
MID	0.330	0.153	2.160	0.031	0.031	0.630
HIGH	0.425	0.157	2.699	0.007	0.116	0.733

a. When the dependent variable is entrepreneurial performance: At low, medium, and high levels of team management ability, the slopes of the impact of risk prevention management ability on entrepreneurial performance are β = 0.130 (*P* = 0.041), β = 0.183 (*P* = 0.005), and β = 0.237 (*P* = 0.001), respectively.

b. When the dependent variable is P&L status: At low and medium levels of team management ability, the slopes of the impact of risk prevention ability on P&L status are not significant (β = 0.046, *P* = 0.698 and β = 0.144, *P* = 0.220, respectively). As team management ability increases to a high level, the impact of risk prevention ability on P&L status becomes significant (β = 0.242, *P* = 0.045).

c. When the dependent variable is the capital flow: At a low level of team management ability, the slope of the impact of risk prevention ability on capital flow is not significant (β = 0.305, *P* = 0.054). With an improvement in team management ability to medium and high levels, the slopes of the impact of risk prevention ability on fund flow both become significant at β = 0.393 (*P* = 0.010) and β = 0.480 (*P* = 0.002), respectively.

d. When the dependent variable is staff flow: At a low level of team management ability, the slope of the impact of risk prevention ability on staff flow (β = 0.236, *P* = 0.128) is not significant. However, when team management ability rises to medium and high levels, the slopes of the impact of risk prevention ability on staff flow become β = 0.330 (*P* = 0.031) and β = 0.425 (*P* = 0.007), respectively, both of which are significant.

To sum up, with an improvement in a team management capacity, risk prevention ability plays an increasingly significant role in the improvement of entrepreneurial performance. When team management ability reaches a medium level, risk prevention ability has a significant impact on capital and staff flows. When team management ability rises to a high level, risk prevention ability plays a role in the P&L status of college students’ new ventures.

## Discussion

### The Impact of Risk Prevention Ability on Entrepreneurial Performance

The impact of risk prevention ability on entrepreneurial performance is significant. In competitive markets, risk prevention ability plays an important role in the stability of an enterprise’s operation. Risks include market risk, external to the enterprise, and management risk, internal to it. Facing various risks in the market, whether an enterprise can effectively identify and predict risks and prepare effective plans and solutions in the face of risks, is an important factor in ensuring the survival and profitability of enterprises. College student entrepreneurs, especially, do not have a deep grasp of the laws of the market and, due to their limited experience, they cannot effectively assess the complexity of the market; thus, their first priority is to ensure a smooth operation of their enterprises. For internal management risks, limited by the lack of management experience, they cannot effectively and comprehensively predict the risks that may be associated with certain company behaviour, which leads to a gradual expansion of the risk coefficient and threatens the stable operation of the enterprises. Preventing and controlling an enterprise’s internal risks are also an important ability for entrepreneurs. With low technology content of products and few market opportunities, whether entrepreneurial enterprises can effectively prevent risks becomes a key factor in their survival.

The results of the model in Table 4 show that the effect of risk prevention capability on entrepreneurial performance is significantly positive and directly acts on P&L status, capital flow, and staff flow. When the model includes team management, risk prevention ability directly impacts staff flow. College students’ new ventures have short-operating times; thus, maintaining the stability of the enterprises is the primary goal to keep the enterprises in continuous operation. The stability of staff flow is a manifestation of the stability of the enterprises, while the financial and fiscal attributes of P&L status and capital flow cannot be reflected in the short period of entrepreneurship. Therefore, the impact of risk prevention ability on entrepreneurial performance is reflected in maintaining the stability of employees, which confirms the pursuit of “survival” of university students’ new ventures.

### The Moderating Role of Team Management Ability

The results of Table 4 and Figures 1–4 show that team management ability positively moderates the impact of risk prevention ability on entrepreneurial performance. College students’ team management ability describes the management of personnel composition, division of labour, and cooperation and communication among team members. People, as implementers of entrepreneurship, are the main input factors of the entrepreneurial process; the structure of core members is directly linked to the success of a start-up. If the core team members are unstable, then the departure of members or the addition of new members is likely to affect the enterprise’s operations. Meanwhile, entrepreneurship is a process of mutual cooperation, rather than one that is completed by the entrepreneur alone. Thus, to ensure the smooth operation of entrepreneurship, it is necessary to ensure division of labour and cooperation, among team members, give full play to their advantages and fulfil their responsibilities, and ensure smooth communication. The stability of the core members of an enterprise indicates team management ability. The cohesiveness of college students’ entrepreneurial teams that proactively accomplish their goals and tasks through a unified ideological understanding, reasonable division of duties, and strict implementation of group decisions will be effective in risk prevention and control in the entrepreneurial process, which in turn will impact the domino effect of entrepreneurial performance. Thus, new ventures with higher levels of team management ability can positively moderate the effectiveness of the impact of risk prevention ability on entrepreneurial performance.

### The “Threshold” Effect of Team Management Ability

The simple slope test in Table 5 and Figure 5 shows that, with a gradual improvement in team management ability, the influence coefficients of risk prevention ability on entrepreneurial performance and various subindicators increase; however, there is an inconsistent “threshold” effect for different performance indicators. When the level of college students’ team management ability is low, risk prevention ability only has a significant effect on entrepreneurial performance, with no significant effect on other indicators. That is, when college students’ entrepreneurial enterprises do not have a certain level of team management ability, risk prevention ability can provide simple support for enterprise performance. With a continuous improvement in team management ability, risk prevention ability plays an increasingly significant role in improving entrepreneurial performance. When team management ability is at a medium level, risk prevention ability can effectively improve capital and staff flows, while when it is at high level, its moderating effect can only play out in the impact of risk prevention on P&L status.

In short, when the level of college students’ entrepreneurial team management ability is low, the entire operating environment for their new ventures is “problem-filled.” Various business problems have negative effects to varying degrees at varying angles through varying links, thus reducing entrepreneurial performance. Therefore, the entrepreneurial team’s risk prevention ability is also weakened or dismembered, and measuring the impact of risk prevention ability on entrepreneurial performance becomes difficult. When a college student’s entrepreneurial company has considerable team management ability, it means that the entrepreneurial team can control the normal operation of the entire enterprise. Under such conditions, the risk prevention ability will directly affect the entrepreneurial performance, ensure that the enterprise has relatively sufficient capital flow, establish a stable entrepreneurial team, and maintain a good profit space for the enterprise. The results show that the higher the team management ability of college students, the stronger the risk prevention ability, and the more significant the performance of college students’ new ventures.

## Conclusion

This study focuses on the impact of college students’ team management ability and risk prevention ability on the performance of their new ventures and verifies the positive effect of risk prevention on entrepreneurial performance as well as the positive moderating effect of team management ability. However, the study has some limitations: it does not consider the impact of factors, such as entrepreneur opportunity identification and enterprise operation on entrepreneurial performance. Further research can be conducted from the perspectives of expanding the factors and improving the entrepreneurial indicators.

This study plays a positive role in guiding the development of college students’ entrepreneurial activities and clarifying the impact of their team risk prevention ability on entrepreneurial performance, including stability and financial performance. The study expounds the moderating effect of team management in promoting enterprise performance and provides ideas for improving the profitability level of Chinese college students’ entrepreneurship. Against the background of China’s deepening economic and social reform and opening-up, it is necessary to cultivate many outstanding entrepreneurs to lead economic development and expand social employability while promoting economic growth. It is necessary to improve college students’ entrepreneurial business performance and the risk prevention management ability of start-up companies. To improve the survival of enterprises, attention must be paid to the establishment of entrepreneurial teams that can effectively drive college students’ entrepreneurial business performance. The results of the study are useful in promoting the development of entrepreneurship education in colleges and universities to attract more college students to participate in entrepreneurial activities and facilitate high-quality development of China’s economy.

## Data Availability Statement

The original contributions presented in the study are included in the article/supplementary material, further inquiries can be directed to the corresponding author.

## Ethics Statement

Ethical review and approval was not required for the study on human participants in accordance with the local legislation and institutional requirements. Written informed consent from the patients/participants was not required to participate in this study in accordance with the national legislation and the institutional requirements.

## Author Contributions

YZ and SQ contributed to conception and design of the study. YZ contributed to data collection, performed the statistical analysis, and wrote the first draft of the manuscript. All authors contributed to the article and approved the submitted version.

## Conflict of Interest

The authors declare that the research was conducted in the absence of any commercial or financial relationships that could be construed as a potential conflict of interest.

## Publisher’s Note

All claims expressed in this article are solely those of the authors and do not necessarily represent those of their affiliated organizations, or those of the publisher, the editors and the reviewers. Any product that may be evaluated in this article, or claim that may be made by its manufacturer, is not guaranteed or endorsed by the publisher.
